# The OMA orthology database in 2018: retrieving evolutionary relationships among all domains of life through richer web and programmatic interfaces

**DOI:** 10.1093/nar/gkx1019

**Published:** 2017-11-02

**Authors:** Adrian M Altenhoff, Natasha M Glover, Clément-Marie Train, Klara Kaleb, Alex Warwick Vesztrocy, David Dylus, Tarcisio M de Farias, Karina Zile, Charles Stevenson, Jiao Long, Henning Redestig, Gaston H Gonnet, Christophe Dessimoz

**Affiliations:** SIB Swiss Institute of Bioinformatics, 1015 Lausanne, Switzerland; ETH Zurich, Computer Science, Universitätstrasse 6, 8092 Zurich, Switzerland; Center for Integrative Genomics, University of Lausanne, 1015 Lausanne, Switzerland; Dept. of Computational Biology, University of Lausanne, 1015 Lausanne, Switzerland; Dept. of Genetics, Evolution & Environment, University College London, Gower St, London WC1E 6BT, UK; Bayer Crop Science NV, Technologiepark 38, 9052 Gent, Belgium; Dept. of Computer Science, University College London, Gower St, London WC1E 6BT, UK

## Abstract

The Orthologous Matrix (OMA) is a leading resource to relate genes across many species from all of life. In this update paper, we review the recent algorithmic improvements in the OMA pipeline, describe increases in species coverage (particularly in plants and early-branching eukaryotes) and introduce several new features in the OMA web browser. Notable improvements include: (i) a scalable, interactive viewer for hierarchical orthologous groups; (ii) protein domain annotations and domain-based links between orthologous groups; (iii) functionality to retrieve phylogenetic marker genes for a subset of species of interest; (iv) a new synteny dot plot viewer; and (v) an overhaul of the programmatic access (REST API and semantic web), which will facilitate incorporation of OMA analyses in computational pipelines and integration with other bioinformatic resources. OMA can be freely accessed at https://omabrowser.org.

## INTRODUCTION

Orthology, the formalization of the intuitive notion of ‘corresponding genes in different species’, is a cornerstone of genomics (reviewed in 1). Two genes are defined as orthologs if they diverged from a common ancestral gene through speciation (2). Orthologs can have conserved biological functions over long evolutionary ranges (e.g. 3) and are thus key to transferring knowledge of biological processes across species. Furthermore, orthologs are used as phylogenetic markers and as anchors to align chromosomes or genomes from different species. Because orthologs are so important, a large number of methods and resources for their inference have been developed over the years, such as the COGs database (4), Inparanoid (5), OrthoMCL (6), Ensembl Compara (7), KEGG Orthology (8), PhylomeDb (9), OrthoDB (10), EggNOG (11), MBGD (12), PLAZA (13) or OMA (14). An overview of general developments in orthology resources are provided in recent reports of the *Quest for Orthologs* consortium (15,16).

OMA (‘Orthologous Matrix’) distinguishes itself through high-quality orthology inferences, a broad coverage of all three domains of life, feature-rich web interface, availability of data in a wide range of formats and interfaces, and a frequent update schedule of two releases per year (14,17).

Here, we present key recent developments of OMA. We first review the improvements in species coverage and in the inference pipeline. Then, we review some of the major new functionalities, including a viewer for hierarchical orthologous groups, domain annotations, a dotplot synteny viewer and improved programmatic accesses. We conclude with a case study of OMA’s use in the industry and with future perspectives.

## SPECIES COVERAGE AND RELEASE SCHEDULE

**Figure 1.**
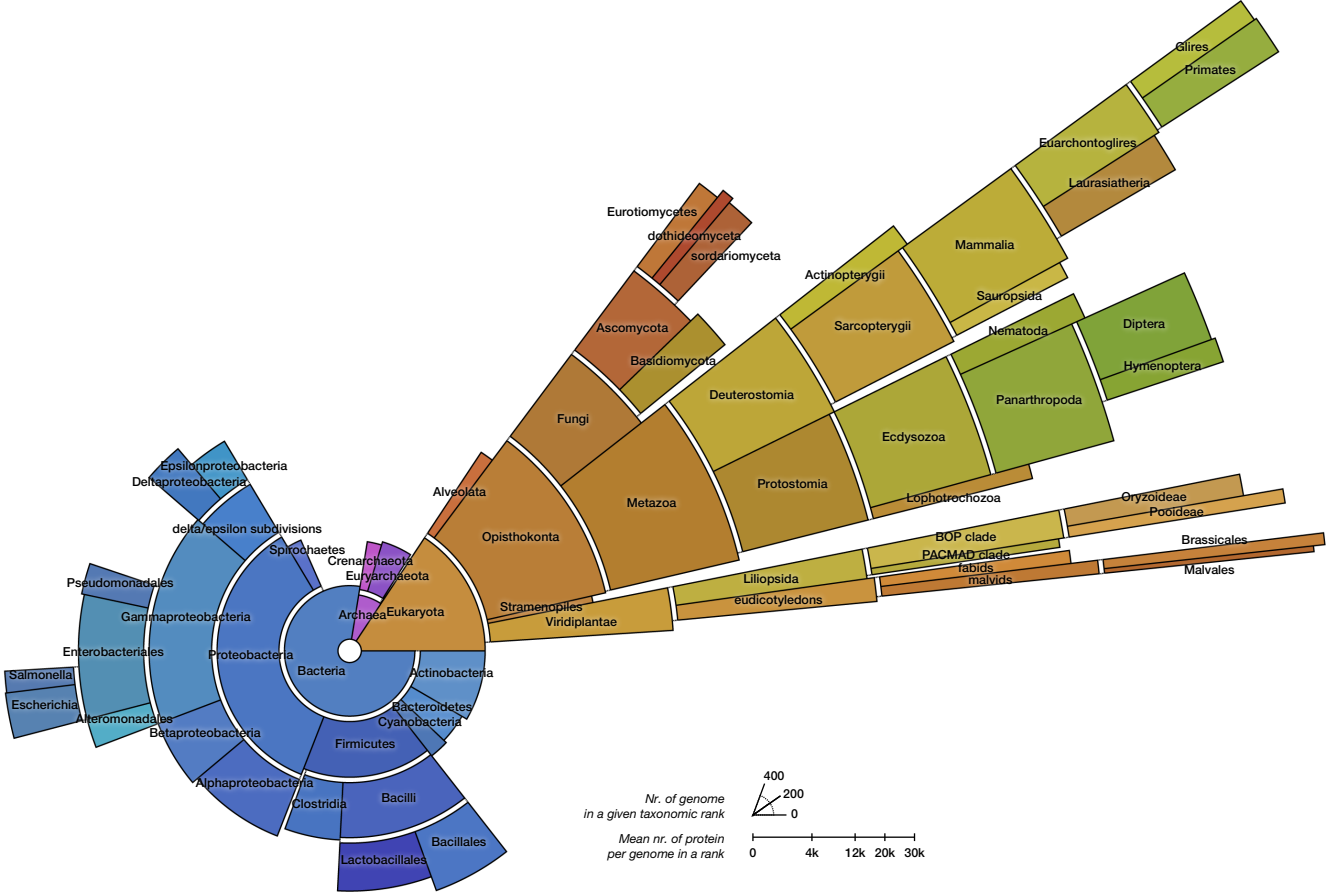
Distribution of the 2085 species contained in the October 2017 OMA release. The number of genomes in each taxonomic rank is conveyed as the angle of the relevant sector, and the average number of proteins is conveyed as its height in a square-root scale. Colors are automatically selected to contrast the different domains of life, and within them the different sister clades.

We strive to release an updated OMA browser two times per year. Since our last update paper (14), there have been five new releases. The newest one covers ∼2100 species with over eleven million protein sequences from all three domains of life (1617 Bacteria, 141 Archaea, 327 Eukaryota; Figure 1). Contrary to most other orthology resources, we also infer orthology across domain boundaries, which makes it possible to identify orthologs shared among e.g. bacteria, archaea, plants, fungi and animals.

In OMA, we update the genomes of the most important model organisms at every release (the 10 genomes with most experimentally backed gene ontology annotations). For other genomes, we only update them if they have been substantially re-annotated. New genomes are generally added to the browser based on user requests, our own needs or that of our collaborators. As a result, we focused our recent efforts on increasing the number of plants, early-branching eukaryotes, drosophila flies and ants. For example, we now cover three allopolyploid plant genomes (bread wheat, rapeseed and upland cotton) and provide homoeology predictions among them (18). OMA users can request new or updated genomes through a web-based form at https://omabrowser.org/suggest. Alternatively, they can still perform their own computations using the OMA standalone software, possibly reusing some of the genomes already analyzed in OMA through the all-against-all export function (14).

## ALGORITHMIC IMPROVEMENTS

From the March 2017 release onward, the OMA Browser uses the updated 2.0 version of the OMA algorithm, which we recently described and benchmarked in a separate publication (19). This new algorithm improves both pairwise orthology and hierarchical orthologous group (HOG) inference. First, it is relatively common, following a gene duplication, for the two copies (‘in-paralogs’) to evolve at different rates. If the duplication occurred within one of two lineages of interest, this induces one-to-many orthologs between them. But because of the asymmetry in the evolutionary rate, one pair may appear to be significantly closer than the other, leading the original OMA algorithm (and other graph-based methods) to only infer the closer one as ortholog—thus missing the other pair. The new version attempts to address this issue by considering the evolutionary distances between in-paralogs, which results in a much higher recall.

Second, we also improved the scalability of HOG inference. We detail the definition and usefulness of HOGs in the next section, but for now it suffices to know that a HOG is a set of genes that have descended from a common ancestral gene in a clade of interest. There is a correspondence between HOGs, gene trees and pairwise orthologs (20). In OMA, we infer HOGs from the pairwise orthologs. The original algorithm, which worked in a ‘top-down’ fashion (from the root of the species tree to the leaves), was too slow to process very large gene families. In OMA 2.0, we introduced a ‘bottom-up’ variant of the algorithm which is several orders of magnitude faster with no negative impact on the performance (19).

## IMPROVED SUPPORT OF HIERARCHICAL ORTHOLOGOUS GROUPS (HOGs)

When simultaneously considering many genomes across all of life, gene families can become huge. This results in complex evolutionary histories consisting of multiple nested evolutionary events. As a result, the traditional approach of considering pairwise relationships or gene trees becomes prohibitively complex to infer and to interpret.

**Figure 2.**
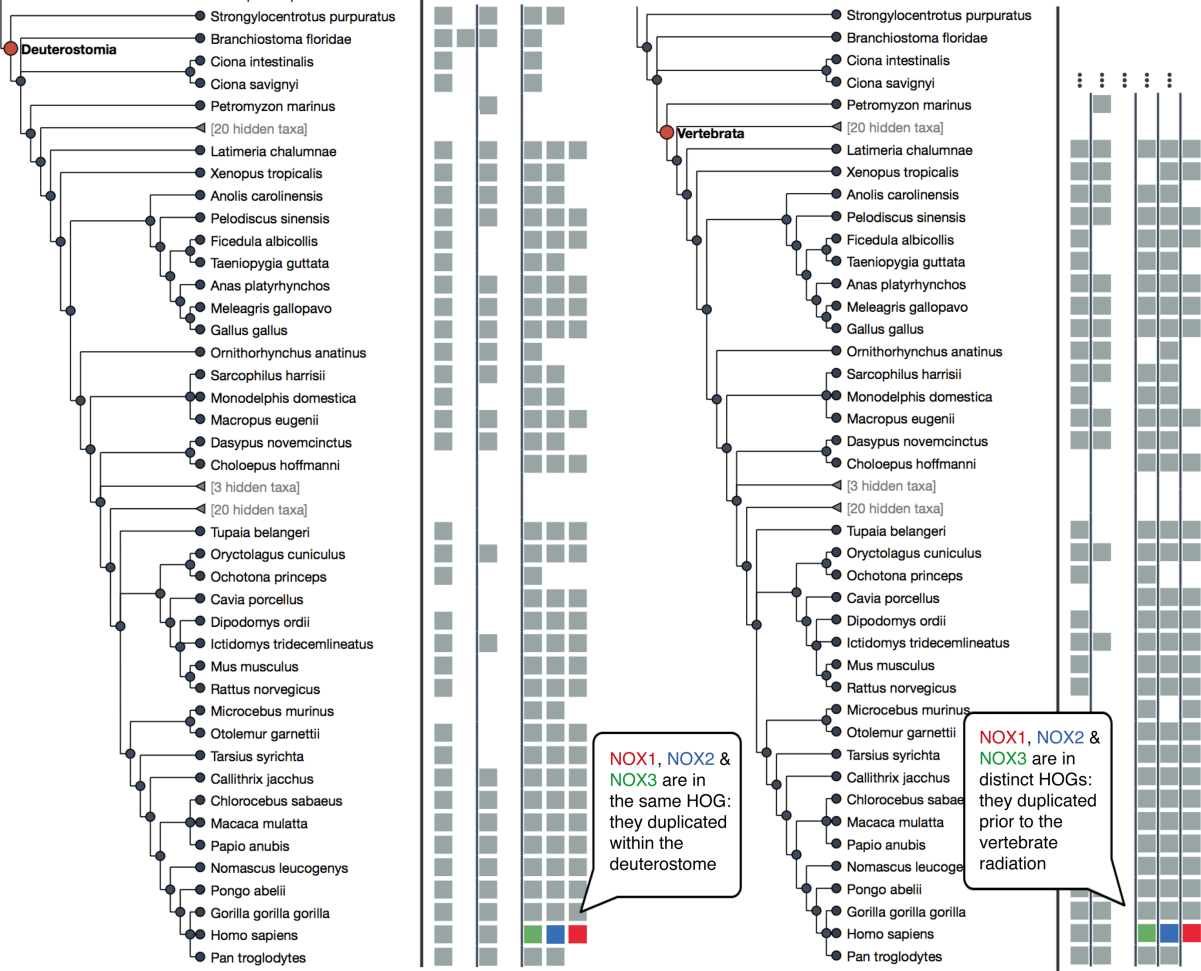
New interactive HOG viewer. An excerpt of the NOX family at the deuterostome level (left) and at the vertebrate level (right). The tree depicts relationships between species, squares depict genes (human NOX1, NOX2 and NOX3 genes are highlighted in color) and HOGs are delineated by vertical black lines.

To make sense of gene evolution in a more scalable framework, OMA adopts the concept of Hierarchical Orthologous Groups (HOGs). HOGs are sets of genes all descendant from a single common ancestral gene within a specific taxonomic range (Figure 2). For instance, the NADPH oxidase (NOX) family in vertebrates contains several paralogs which result from gene duplications, mostly ancestral to the vertebrates (21,22). Although their general sequence, structure, and function is relatively well conserved, the paralogous copies are associated with different diseases, indicating subtle but important differences among the copies (23). At the vertebrate taxonomic level, NOX1, NOX2 and NOX3 genes are clustered by OMA into distinct HOGs, consistent with the accepted notion that these were already distinct copies in the last common ancestor of the vertebrates. By contrast, at the Deuterostome taxonomic level, the three copies are clustered in the same HOG, indicating that they descended from a single ancestral gene in the last common ancestor of the Deuterostomes. Thus duplication of these genes is likely to have occurred in between the deuterostomes and vertebrate branches in the tree of life—perhaps as part of the 2R whole genome duplication at the basis of the vertebrates (24).

We now provide a HOG viewer in OMA, which takes advantage of the interactive and dynamic nature of modern web widgets. The viewer is composed of a familiar species tree, which lets the user select the taxonomic range of interest by clicking on the corresponding ancestral node, highlighted in red (Figure 2). Right of the tree, the viewer displays extant genes as squares, horizontally aligned with the species to which they belong. Crucially, genes are partitioned in HOGs according to the taxonomic level of reference, where HOG boundaries are denoted by vertical bars. It is possible to color the genes according to the corresponding protein lengths or GC content. Furthermore, it is also possible to remove HOGs that only contain a low proportion of genes across the taxonomic range of interest, because many of these are likely to be spurious. The viewer is implemented using the flexible TNT javascript framework (25).

We have also improved HOGs data structure retrieval for user-side analysis. HOG pages now feature dynamic tables with a domain architecture viewer. Individual HOG datums, such as the HOG structure in OrthoXML format (26), or a fasta file of the sequences for all the genes contained in that HOG, are now available for download directly from the OMA Browser (see also section below on programmatic access). In addition, we have recently developed a standalone python package (‘pyham’) which can be used to retrieve either single HOGs, or patterns of gene duplications and losses for multiple HOGs. Pyham can be installed by the standard Python package manager ‘pip’.

## DOMAIN ANNOTATIONS AND EXPLORATION

OMA now integrates domain annotations from Gene3D for individual protein entries (27). Currently, 78.3% of all entries in OMA have a domain annotation, resulting in an overall proportion of 55.1% amino-acid residues annotated as part of a domain. For each protein, the sequence of annotated domains is depicted using the conventional ‘colored-boxes-on-a-line’ representation, which we include in most protein lists. This makes it possible to easily check whether the domain architecture of a protein is conserved among orthologs, or to identify entries which are likely to be truncated or otherwise problematic. CATH domains (28) are depicted in colors specific to their first and second level classification. We assign the most prevalent domain architecture to the HOG itself.

Domains can also be used to establish links between HOGs. Given an initial HOG, a user can retrieve a table of the most similar HOGs based on conserved domain architecture. The similarity is computed by counting the number of domains in common between two HOGs. Genes that belong to distinct but similar HOGs can be paralogs separated by a very deep duplication, orthologs misclassified by OMA in separate groups or genes that are homologous for only part of their sequence (e.g. genes spanning over a domain fusion or fission event, artefactual fragments, etc.). This domain architecture view allows users to estimate how specific or widespread the domains that make up a protein family are, and allows them to make hypotheses about the origin of a protein family.

**Figure 3.**
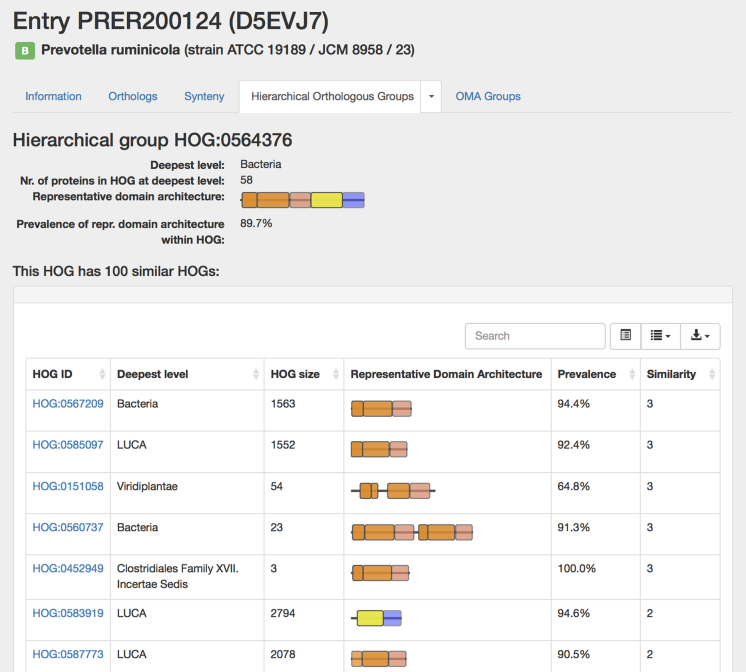
The domain architecture view of a HOG. Information about the HOG (on the top) is followed by the table containing information about other HOGs that share at least one domain in common with the HOG of interest. Deepest level: the last common ancestor of the species represented in a HOG; HOG size: the number of genes in a HOG; Representative Domain Architecture: the architecture that is characteristic of most of the proteins in a HOG; Prevalence: the percentage of the proteins in a HOG that have this domain architecture; Similarity: the number of the domains shared between this HOG and the HOG of interest (including duplicated domains). The table can be sorted by any of the attributes.

For example, Figure 3 depicts a ligase family specific to Bacteria (HOG:0564376) that could have originated from a fusion of a ubiquitous ligase family (HOG:0585097) with Carboxynorspermidine decarboxylase enzyme family (HOG:0580230). The domain-based search also identifies the Bacteria-specific family of UDP-N-acetylmuramyl-tripeptide synthetase (HOG:0560737), which is likely to have originated from a tandem duplication of a member of the ubiquitous ligase family.

## PHYLOGENETIC MARKER GENE EXPORT

To infer a phylogenetic species tree, it is first necessary to identify sets of orthologous genes among the genomes of interest. One of the outputs of the OMA database are ‘OMA Groups,’ or sets of genes which are all orthologous to each other. Since genes in OMA Groups are related exclusively by speciation events, there is at most one sequence per species in each OMA group. In contrast to most other phylogenetic methods, OMA makes no assumption about species relationships when inferring OMA groups. This makes OMA Groups particularly useful for phylogenetic species tree inference.

The OMA groups are computed at each release over all species. Since many users are only interested in a small subset of genomes, we now provide a function to retrieve, for a given subset of species, the most complete OMA groups. The new functionality, entitled ‘Export marker genes’, is accessible under the ‘Compute’ menu. Users can optionally choose a minimum proportion of species present in each group (‘occupancy’), and a maximum number of groups to export. From the choice of species and parameters, the OMA server identifies the most complete groups and produces a compressed archive file containing one fasta file per marker gene (i.e. per OMA group).

To illustrate this functionality, we exported marker genes for all 88 Fungi in the March 2017 release, requesting 100 markers with at least 50% occupancy. We independently aligned each group using Mafft (29), concatenated the resulting alignments without filtering (30) and inferred trees using FastTree (31)—using default parameters of each software tool. The entire procedure took 40 minutes on a single CPU, mostly spent aligning sequences. The resulting tree, highly resolved, is congruent with the NCBI taxonomy, with the sole exception of the placement of *Fomitopsis pinicola* (the disagreeing branch has however a lower support of 0.84; Supplementary Figure S1).

## SYNTENY DOTPLOT

When comparing two related species, the position of orthologous genes is often conserved. Positional conservation can be at the chromosomal level—e.g. when there are entire chromosomes or chromosomal segments that are orthologous between species; or it can be more local—e.g. neighboring genes in one genome are orthologous to neighboring genes in the other genome. In OMA, we refer to global synteny for the former, and local synteny for the latter (local synteny is sometimes also referred to as ‘colinerarity’).

The breakdown of synteny can be caused by gene movement via transposition/translocation, as well as large chromosomal or segmental rearrangements. Conservation of synteny, or lack thereof, can have several uses and implications in evolutionary and comparative genomics: for example, synteny can be used to gauge how closely related genomes are, to identify genomic rearrangements, to reconstruct ancestral genomes and to aid genome assembly.

A few years ago, we introduced a local synteny viewer in OMA, which enables users to see orthology of neighboring genes across many species (14). This functionality has proven useful, particularly if we consider that many gene duplications are tandem duplication, and thus one-to-many and many-to-many orthology relationships can often be depicted even if one focuses on a narrow genomic window in each species. However, to identify larger events, such as large duplications and inversions, or to identify non-syntenic orthologs between an otherwise largely syntenic pair of genomes, a more global view is necessary.

**Figure 4.**
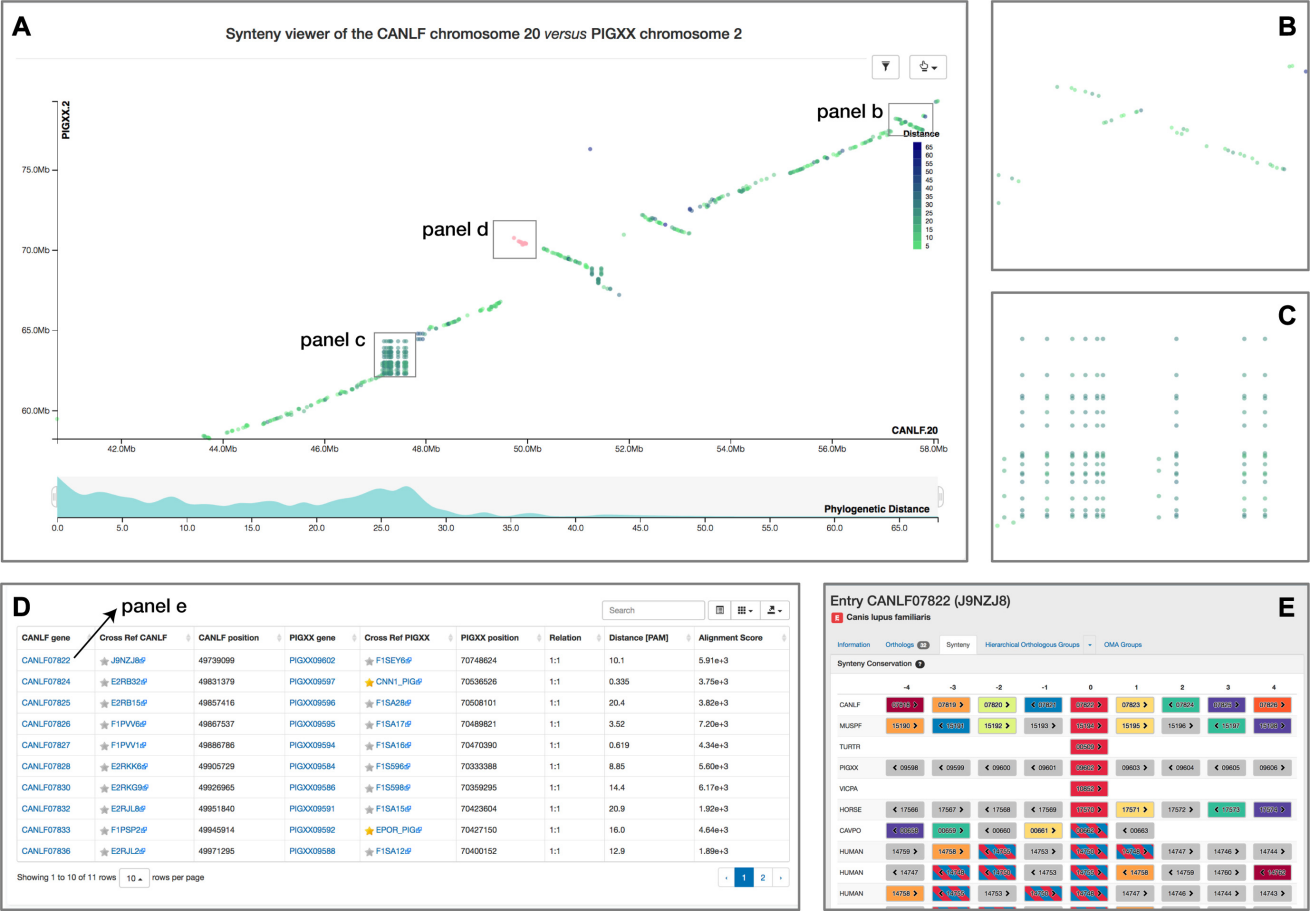
New dotplot synteny viewer, which enables users to identify gene order conservation between chromosomes as diagonal segments (main view in panel **A**). Inversions are visible as diagonal flips, which can be nested (panel **B**). Tandem duplications on one or the other chromosome are visible as vertical or horizontal lines—and, if both are present, as blocks (panel **C**). To focus on a subset of the data according to sequence divergence, the user can restrict the desired range of the distribution of the evolutionary distance of each point. Points can be selected by the user, in which case more details are provided in a table (panel **D**), including links to the local synteny viewer (panel **E**).

Here, we introduce a synteny dotplot viewer in the OMA Browser. For any pair of chromosomes (in different species if we consider orthologs, or different subgenomes if we consider homoeologs), the plot draws orthologs as dots on a two-dimensional plot, where the axes are absolute physical location of the genes along the chromosome. Diagonals in the plot can thus be interpreted as syntenic regions, and one can easily identify genomic rearrangements such as inversions, duplications, insertions, deletions and highly repetitive regions (Figure 4). Users can zoom on particular regions of interest and obtain more details on orthologs of interest by selecting them. Each dot is colored based on a color scale reflecting the evolutionary distance in point accepted mutation (PAM) units. Furthermore, one can filter the orthologs to a specific distance range by clicking on the filtering icon and selecting the desired range on a histogram. Other features include panning and exporting the view as a high-resolution vector graphic. Thus, the new synteny dotplot complements the existing local synteny viewer by providing a more global and interactive view of positional conservation.

## GO FUNCTION ANNOTATIONS

An important application of orthology is the ability to transfer gene function annotations from the few well-studied model organisms to the large number of poorly studied genomes. We previously described our approach to predict Gene Ontology (GO) annotations from OMA Groups (14). The approach was found to perform well in the Critical Assessment of Function Annotation 2 (CAFA2) experiment (32), where it scored highly under several criteria. Note however that large-scale benchmarking of functional prediction is notoriously difficult (33), so these results should be interpreted with caution.

In the same spirit as the mapping tool of the EggNOG database (34), we now provide a feature to annotate custom protein sequences through a fast approximate search with all the sequences in OMA. The user can upload a fasta formatted file and will receive the GO annotations (GAF 2.1 format) based on the closest sequence in OMA. These results can directly be further analyzed using other tools, e.g. to perform a gene enrichment analysis (reviewed in 35). This functionality is accessible under the ‘Compute’ menu in the OMA browser.

## MODERN PROGRAMMATIC ACCESS: REST AND SPARQL

Allowing users to programmatically query the OMA data has been a goal early on: in 2007 we introduced Simple Object Access Protocol (SOAP) API and Distributed Annotation Service (DAS) endpoints. Since then, both technologies have however fallen out of favor by many users or developers. We are thus discontinuing support for SOAP and DAS, and replacing them with new Representational State Transfer (REST) and SPARQL Protocol and RDF Query Language (SPARQL) APIs.

The new REST API provides programmatic access to a comprehensive set of features provided through the web server. This API can be used to automate almost any analysis that a user could do on the website. On the REST API documentation page, which is accessible under https://omabrowser.org/api, all the endpoints and their parameters are described. Each endpoint includes also a live example. In addition, for R and python users, we provide native libraries wrapping around the REST API that further facilitates querying the OMA database in these languages.

**Figure 5.**
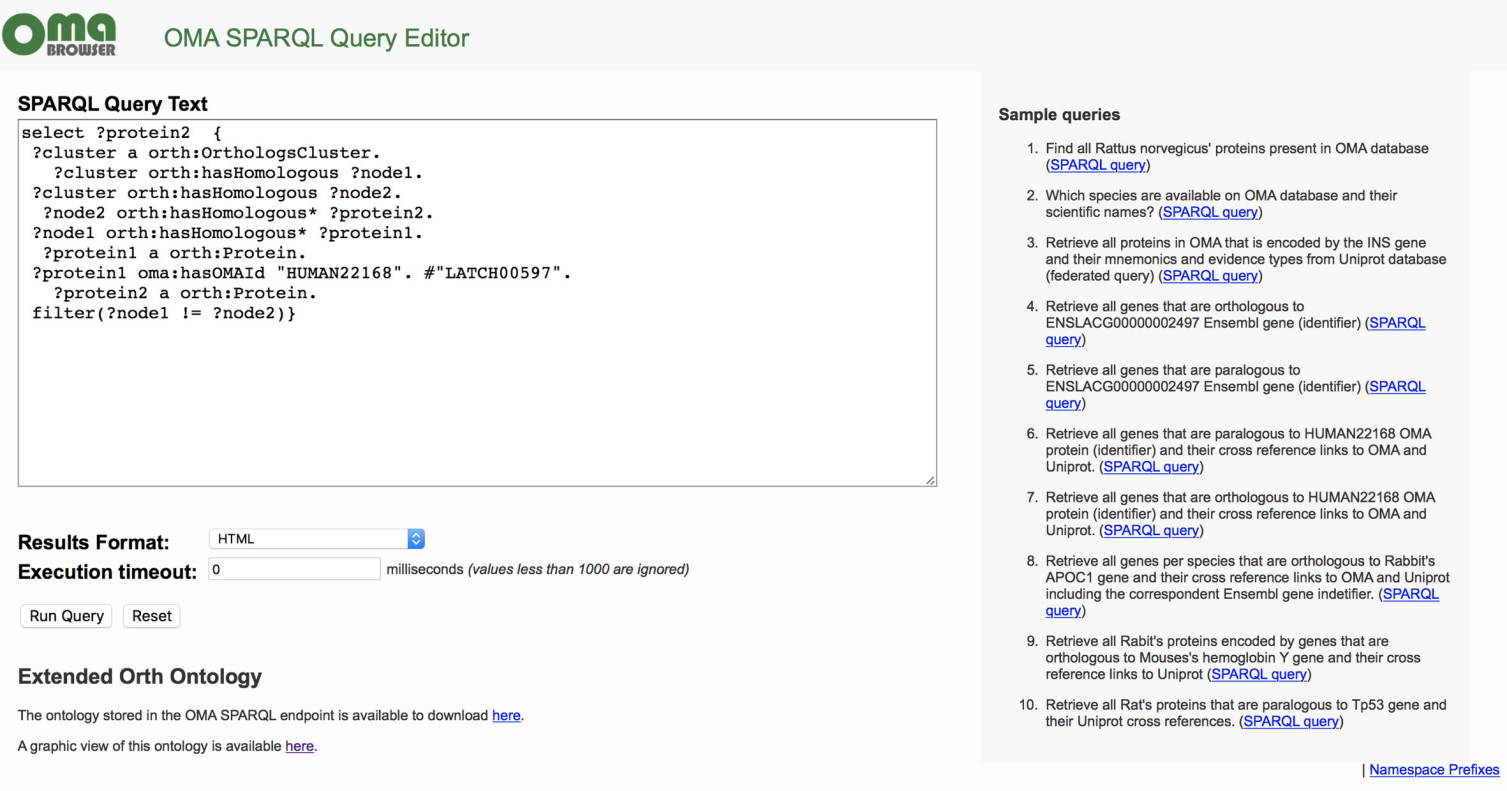
Example of a SPARQL query to programmatically retrieve pairwise orthologs involving the sequence LATCH00597. Sample queries are provided in the right column of the page, accessible at http://sparql.omabrowser.org.

Ontologies provide a way to describe and organize concepts used in biological databases, and thereby facilitate data interoperability across multiple resources. An Orthology Ontology (ORTH) was recently introduced (36), and we adapted and extended the ORTH ontology to fully support OMA. To enhance interoperability among resources, this updated ontology uses whenever possible terms compatible with other resources, such as the Microbial Genome Database (MBGD) (12) and Universal Protein Resource (UniProt) (37) ontologies. This version also describes additional orthology data such as OMA groups, domain architecture, nucleotide sequences and cross-references. Moreover, one of the major interoperability issues of orthology and life science databases is the heterogeneity of gene and protein identifiers used in these databases. To solve this issue, we extended the ORTH ontology by defining terms to explicitly represent multiple gene and protein identifiers such as the OWL property *identifier* and its sub-properties *ensemblGeneId, uniProtId, entrezGeneId* and *hasOMAId*. Therefore, these terms can be used by other data providers to avoid ambiguity among different identifiers. Furthermore, based on this extended version of the ORTH ontology, we released a SPARQL endpoint that is available on https://sparql.omabrowser.org to compose complex and federated queries over orthology and life science data (Figure 5).

## OTHER NOTEWORTHY IMPROVEMENTS TO THE WEB INTERFACE

In addition to the above, we have implemented a number of smaller refinements that are worth mentioning here.

We now use dynamic tables for most lists in OMA. This enables users to sort according to the various table columns and to search rows using keywords. Responsiveness is also improved, with asynchronous loading of the table content and flexible pagination of the results. Finally, the new interface makes it easier to export the table contents in a variety of formats (e.g. JSON, XML, CSV, etc.).

The search function in OMA now supports autocompletion of identifiers and gene names. Whenever available, we use the gene name established by the HUGO gene nomenclature committee (38).

To display multiple sequence alignments, which we compute both for HOGs and OMA groups using Mafft (29), we now use the native web viewer MSAviewer (39).

We have also streamlined communication with users. OMA users can follow our latest updates on Twitter (@omabrowser), following the OMA blog (http://omabrowser.blogspot.com) or by signing up to our low frequency mailing list oma@lists.dessimoz.org. If they have questions, the preferred way to reach us is by asking questions on the BioStars Q&A platform (40) using the tag ‘oma’.

The species selection in the all-against-all export functionality now uses the phylo.io tree viewer (41). All basic features of manipulation of a phylogenetic tree are included, such as label searching, re-rooting or branch swapping. Selected species are now automatically highlighted, making it easier to keep an overview on the tree of what is selected for export. Finally, once the final list of exported species is selected, phylo.io allows users to trim residual branches and display the tree of selected species only.

## USE OF OMA IN THE INDUSTRY: THE EXAMPLE OF BAYER CROP SCIENCE

The access to accurate orthology relationships across all relevant species provides added value for applied research in industry applications, particularly at plant biotechnology companies. OMA collaborates with Bayer Crop Science (BCS) to accelerate the process of discovering and validating genes associated with crop traits related to yield potential, maintenance and tolerance to biotic and abiotic stresses by enabling the efficient mapping of gene functional information across model and crop species.

Through the five-year collaboration, we have deployed a private, scalable and extensible OMA instance combining proprietary and publicly available genomes from plant, insect, fungal and microbial species. Together with PLAZA (13), it constitutes the comparative genomics framework enabling BCS scientists to query orthologous pairs, to visualize the diversity in genomic content, to study the phylogenetic profiles of gene families of interest and to perform computational functional annotation based on orthology relationships.

The OMA@BCS resource is also updated twice a year, in line with the public OMA. The build code is merged to BCS code repositories on a regular basis and the publicly integrated data can be reused by BCS without repeating the computationally intensive all-against-all alignments thanks to the permissive licensing policy of the OMA project (Creative Common BY-SA 2.5 for the web browser, and open source MLP 2.0 license for code).

## FUTURE PERSPECTIVES

This paper surveys a substantial number of improvements to the algorithm, coverage and interfaces of the OMA database. Just as importantly, OMA continues to be maintained and regularly updated.

As the cost of sequencing continues to drop, genomic data is shifting from consortium-led, general purpose, sequencing efforts to one-off user-generated data. OMA is adapting accordingly. We will continue to provide up-to-date, high-quality and user-friendly orthology relationships among many genomes across all of life in the public OMA database; in so doing, we will prioritize general-purpose, high-quality genomes, with a special effort toward better sampling life's diversity. At the same time, through web services operating on user-submitted data (e.g. the new function prediction tool introduced above), more flexible programmatic access, and OMA standalone, we aim to facilitate orthology analyses on custom data. And as our collaboration with Bayer demonstrates, it is already possible to deploy custom OMA Browser instances within organizations or individual laboratories interested in relating in-house data.

## Supplementary Material

Supplementary Data
