# Supplementary material for: The OMA orthology database in 2018: retrieving evolutionary relationships among all domains of life through richer web and programmatic interfaces
Source: Nucleic Acids Res. 2017 Nov 2;46(Database issue):D477–85. doi: 10.1093/nar/gkx1019 (PMC5753216; doi:10.1093/nar/gkx1019)
Supplement: Supplementary Data [file gkx1019_supp.pdf]

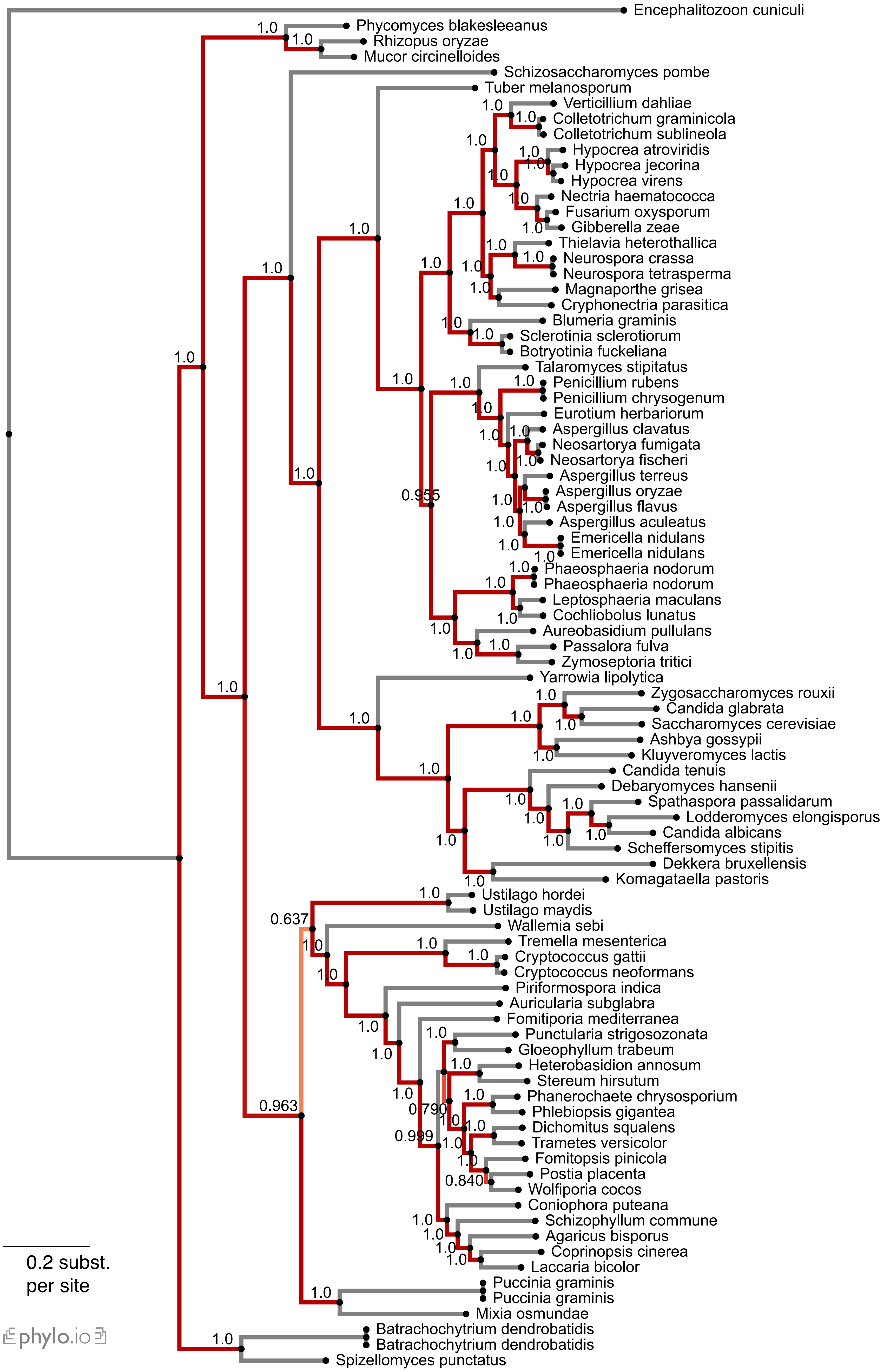

Supplementary figure 1: Fungi tree inferred from 100 orthologous groups exported using the new “marker gene export” function. The tree, which could be computed within 40 minutes on a single CPU, is highly congruent with, but considerably more resolved than the NCBI taxonomy.
